# Serious adverse events and fatal adverse events associated with nivolumab treatment in cancer patients: Nivolumab-related serious/fatal adverse events

**DOI:** 10.1186/s40425-018-0421-z

**Published:** 2018-10-03

**Authors:** Bin Zhao, Hong Zhao, Jiaxin Zhao

**Affiliations:** 1 The Second Affiliated Hospital & Yuying Children’s Hospital, Wenzhou Medical University, Wenzhou, China; 2 The Third Affiliated Hospital of Harbin Medical University, Harbin, China; 3 The Fourth Affiliated Hospital of Harbin Medical University, Harbin, China

**Keywords:** Serious adverse events, Fatal adverse events, Cancer, Nivolumab

## Abstract

**Background:**

Nivolumab, an immune checkpoint inhibitor, has revolutionized the treatment of many cancers. Due to its novel mechanisms of action, nivolumab induces a distinct profile of adverse events. Currently, the incidence and risk of developing serious adverse events (SAEs) or fatal adverse events (FAEs) following nivolumab administration are unclear.

**Methods:**

We conducted a systematic search for phase 2 and phase 3 nivolumab trials in PubMed and Embase from inception to June 2018. Data on SAEs/FAEs were extracted from each study and pooled to calculate the overall incidence and odds ratios (ORs).

**Results:**

A total of 21 trials with 6173 cancer patients were included in this study. The overall incidence of SAEs and FAEs with nivolumab were 11.2% (95% CI, 8.7–13.8%) and 0.3% (95% CI, 0.1–0.5%), respectively. The incidence of SAEs varied significantly with cancer type and clinical phase, but no evidence of heterogeneity was found for FAEs. Compared with conventional treatment, the administration of nivolumab did not increase the risk of SAEs (OR, 0.69; 95% CI, 0.34–1.40; *p* = 0.29) or FAEs (OR, 0.61; 95% CI, 0.27–1.39; *p* = 0.24). SAEs occurred in the major organ systems in a dispersed manner, with the most common toxicities appearing in the respiratory (21.4%), gastrointestinal (7.7%), and hepatic systems (6.6%). The most common cause of SAEs/FAEs was pneumonitis.

**Conclusions:**

Although nivolumab is a relatively safe antitumor agent, nononcologists should be advised of the potential adverse events. Additionally, future studies are needed to identify patients at high risk of SAEs/FAEs to aid in the development of optimal monitoring strategies and the exploration of treatments to decrease the risks.

## Background

Immune suppression and evasion of malignant cancer cells are hallmarks of cancer [1]. A series of coinhibitory and costimulatory receptors and their ligands, known as immune checkpoints, control these processes. Among them, the programmed cell death protein 1(PD-1)/programmed death-ligand 1(PD-L1) axis stands out as a valuable therapeutic target [2]. The development and application of antibodies targeting PD-1 and PD-L1 have been major advances in cancer treatment [3]. Nivolumab, a fully human IgG4 monoclonal antibody against PD-1, has been approved for the treatment of many cancers [3].

It is well known that cancer treatment is usually a double-edged sword. Patients often tend to overestimate the benefit and to underestimate the disadvantages [4]. SAEs can lead to treatment discontinuation, hospitalization, or even death. Accordingly, information on SAE/FAE prevalence should play an important role in the decision-making process in clinical practice. Without fully understanding the risks of SAE/FAE, healthcare practitioners and patients cannot properly balance the benefits and risks. Previous work has shown that ipilimumab, an immune checkpoint inhibitor targeting cytotoxic T-lymphocyte-associated protein 4 (CTLA-4), was associated with significantly increased risks of treatment-related mortality [5]. Both nivolumab-related SAEs and FAEs have been reported in clinical practice [6–12] and have unfortunate consequences for the patients. Accordingly, it is important to fully investigate the toxicities related to this agent. However, the incidences and risk of SAEs/FAEs are often overlooked, partly due to the lack of data. The expanding application of nivolumab for the treatment of various cancers clearly necessitates rigorous and reliable information for evaluating nivolumab-related SAEs/FAEs. Moreover, as the use of nivolumab grows, nononcology specialists will be increasingly called upon to manage the rare but clinically important organ-specific adverse events and the more prevalent general adverse events related to immune activation. Based on accumulating evidence, our aim was to summarize nivolumab-related SAEs/FAEs and to estimate the incidence and risk of SAEs/FAEs by conducting a meta-analysis among adult patients with solid tumors.

## Method

### Search strategy

A systematic search of the Embase and PubMed databases from inception to June 2018 was conducted with no language restrictions. Conference proceedings from the American Society of Clinical Oncology and the European Society for Medical Oncology were also searched. The search keywords and medical subject headings used were (1) Nivolumab, Opdivo, ONO-4538, BMS-936558 and MDX1106; and (2) clinical trial. Three investigators independently performed the initial search, carefully screened the titles and abstracts for relevance, and identified trials as excluded, included and uncertain. For the uncertain studies, the full-texts were reviewed for the confirmation of eligibility. Any discrepancy was resolved by discussion.

### Eligibility criteria

**Fig. 1:**
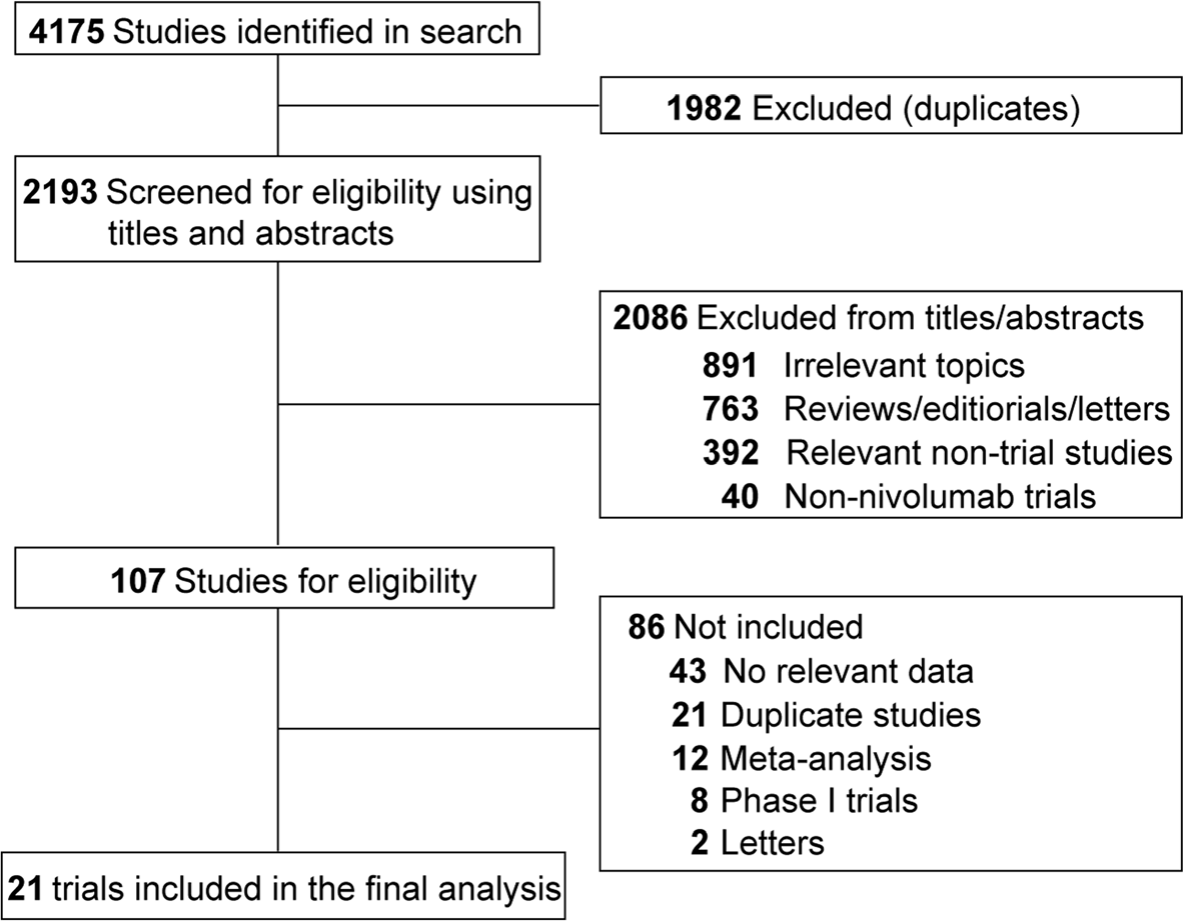
Flow chart of the eligible trials included in this study

Both the inclusion and exclusion criteria were prespecified. To be eligible, studies had to meet the following criteria: (1) population: clinical phase 2 and phase 3 prospective trials involving adult patients (> 18 years old) with solid tumors; (2) intervention: at least one arm with nivolumab monotherapy; and (3) outcomes: available information on sample size, SAEs and FAEs. Phase I trials were not included because of the small sample size of patients and various doses in these studies. Other studies on this topic, including review articles, preclinical papers, early versions of data published later, editorials, and correspondences, were not included (Fig. 1). When multiple publications of the same study occurred, only the most recent and/or most complete study was included.

### Data extraction

An FAE is defined as death caused by nivolumab treatment, and an SAE refers to any adverse event (AE) that may lead to death, hospitalization or the prolongation of hospitalization, a life-threatening condition, congenital anomaly or birth defect, disability or permanent damage, or jeopardization of the patient’s health in a way that requires treatment to prevent one of the other outcomes. The attribution of FAEs or SAEs as treatment-related or disease-related depended on the decision of the primary investigators in the eligible studies. The information on ‘treatment-related SAE’ and ‘treatment-related FAE’ was extracted from the original publications. For all of the eligible studies, we also extracted the following information: first author’s name, year of publication, phase of the trial, cancer type, number of patients enrolled, median age, gender, treatment strategy, median treatment duration and median follow-up (Table 1). In addition, the median overall survival (OS) in each treatment arm, the hazard ratio (HR) for the OS, the number of participants evaluated for safety, the number of FAEs, and the number of SAEs were listed in Table 2. All data were extracted independently by three reviewers, and any discrepancies were resolved by discussion and consensus.

**Table 1:** Clinicopathological characteristics of trials included in this study

Study	Trial phase	Underlying malignancy	No. of patients enrolled	Median age (range), year	Gender (Male/ Female)	Treatment	Median treatment duration (range), month	Median follow-up (range), month
Borghaei,2015 [6]	3	Lung cancer	292	61(37–84)	151/141	Nivolumab 3 mg/kg every 14 days	3.0(0.5–26.0)	> 13.2
			290	64(21–85)	168/122	Docetaxel 75 mg/m^2^ every 21 days	3.0(0.8–17.3)	
Brahmer, 2015 [7]	3	Lung cancer	135	62(39–85)	111/24	Nivolumab 3 mg/kg every 14 days	4.0(0.5–24.0)	< 11.0
			137	64(42–84)	97/40	Docetaxel 75 mg/m^2^ every 21 days	2.3(0.8–21.8)	
Carbone, 2017 [8]	3	Lung cancer	271	63(32–89)	184/87	Nivolumab 3 mg/kg every 14 days	3.7(0–26.9)	13.5
			270	65(29–87)	148/122	Chemotherapy once every 21 days	3.4(0–20.9)	
Ferris, 2016 [10]	3	Head and neck cancer	240	59(29–83)	197/43	Nivolumab 3 mg/kg every 14 days	1.9	5.1(0–16.8)
			121	61(28–78)	103/18	Systemic therapy single-agent	1.9	
Kang, 2017 [9]	3	G/GJC	330	62(54–69)	229/101	Nivolumab 3 mg/kg every 14 days	1.9	8.9
			163	61(53–68)	119/44	Placebo 3 mg/kg every 14 days	1.9	8.6
Motzer, 2015 [11]	3	Renal cancer	410	62(23–88)	315/95	Nivolumab 3 mg/kg every 14 days	5.5(0–29.6)	> 14.0
			411	62(18–86)	304/107	Everolimus 10 mg daily	3.7(0.2–25.7)	
Robert, 2015 [12]	3	Melanoma	210	64(18–86)	121/89	Nivolumab 3 mg/kg every 14 days	NR	< 16.7
			205	66(26–87)	125/83	Dacarbazine 1 g/m^2^ every 21 days	NR	
Weber, 2017 [13]	3	Melanoma	453	56(19–83)	258/195	Nivolumab 3 mg/kg every 14 days	12.0	> 18.0
			453	54(18–86)	269/184	Ipilimumab 10 mg/kg every 21 days	3.0	
Wolchok, 2017 [14]	3	Melanoma	314	61(18–88)	206/108	Nivolumab 1 mg/kg + ipilimumab 3 mg/kg every 21 days	6	38
			316	60(25–90)	202/114	Nivolumab 3 mg/kg every 14 days	7.5	35.7
			315	62(18–89)	202/113	Ipilimumab 3 mg/kg every 21 days	3	18.6
D’angelo, 2018 [15]	2	Sarcoma	43	56(21–76)	22/21	Nivolumab 3 mg/kg every 14 days	2.3	13.6
			42	57(27–81)	19/23	Nivolumab 3 mg/kg + ipilimumab 1 mg/kg every 21 days	3.7	14.2
Long, 2018 [16]	2	Melanoma	35	59(53–68)	29/6	Nivolumab 1 mg/kg + ipilimumab 3 mg/kg every 21 days	NR	14.0
			25	63(52–74)	19/6	Nivolumab 3 mg/kg every 14 days	NR	17.0
			16	51(48–56)	11/5	Nivolumab 3 mg/kg every 14 days	NR	31.0
Motzer, 2015 [19]	2	Renal cancer	60	61	41/19	Nivolumab 0.3 mg/kg every 21 days	4.5(0–21.8)	> 24.0
			54	61	40/14	Nivolumab 2 mg/kg every 14 days	5.6(0.8–24.0)	
			54	61	40/14	Nivolumab 10 mg/kg every 14 days	6.0(0.8–23.3)	
Hamanishi, 2015 [18]	2	Ovarian cancer	20	60(47–79)	0/20	Nivolumab 1 or 3 mg/kg every 14 days	3.5(1.0–12.0)	11.0(3.0–32)
Hida, 2017 [29]	2	Lung cancer	35	65(31–85)	32/3	Nivolumab 3 mg/kg every 14 days	3.6(0.5–29.3)	< 30.0
Kudo, 2017 [30]	2	Esophageal cancer	65	62(49–80)	54/11	Nivolumab 3 mg/kg every 14 days	105(0.5–5.0)	10.8
Maruyama, 2017 [31]	2	Hodgkin lymphoma	17	63(29–83)	13/4	Nivolumab 3 mg/kg every 14 days	7.0(1.4–10.6)	9.8(6.0–11.1)
Nishio, 2017 [32]	2	Lung cancer	76	64(39–78)	49/27	Nivolumab 3 mg/kg every 14 days	NR	16.6(0.9–31.9)
Overman, 2017 [33]	2	Colorectal cancer	74	53(44–64)	44/30	Nivolumab 3 mg/kg every 14 days	11.0	12.0
Rizvi, 2015 [34]	2	Lung cancer	117	65(57–71)	85/32	Nivolumab 3 mg/kg every 14 days	2.3	8.0
Yamazaiki, 2017 [35]	2	Melanoma	24	63(26–81)	14/10	Nivolumab 3 mg/kg every 14 days	11.9(0.5–21.0)	18.8(2.0–21.5)
Younes, 2016 [36]	2	Hodgkin lymphoma	80	37(28–48)	51/29	Nivolumab 3 mg/kg every 14 days	8.5	8.9

*Abbreviation: G/GJC* gastric and gastro-esophageal junction cancer

**Table 2:** The risk and benefit of nivolumab treatment in cancer

Study	Underlying malignancy	No. of patients enrolled	Median OS (95% CI), month	HR (95% CI)	*P* value	No. of patients (safety)	FAE	SAE
Borghaei,2015 [6]	Lung cancer	292	12.2(9.7–15.0)	0.73(0.59–0.89)	0.002	287	1	21
		290	9.4(8.1–10.7)			268	1	53
Brahmer, 2015 [7]	Lung cancer	135	9.2(7.3–13.3)	0.59(0.44–0.79)	< 0.001	131	0	9
		137	6.0(5.1–7.3)			129	3	31
Carbone, 2017 [8]	Lung cancer	271	14.4(11.7–17.4)	1.02(0.80–1.30)	NR	267	2	46
		270	13.2(10.7–17.1)			263	3	48
Ferris, 2016 [10]	Head and neck cancer	240	7.5(5.5–9.1)	0.70(0.51–0.96)	0.01	236	2	NR
		121	5.1(4.0–6.0)			111	1	NR
Kang, 2017 [9]	G/GJC	330	5.3(4.6–6.4)	0.63(0.51–0.78)	< 0.0001	330	5	33
		163	4.1(3.4–4.9)			161	2	8
Motzer, 2015 [11]	Renal cancer	410	25.0(21.8-not reached)	0.73(0.57–0.93)	0.002	406	0	NR
		411	19.6(17.6–23.1)			397	2	NR
Robert, 2015 [12]	Melanoma	210	Not reached	0.42(0.25–0.73)	< 0.001	206	0	19
		205	10.8(9.3–12.1)			205	0	18
Weber, 2017 [13]	Melanoma	453	NR	NR	NR	452	0	NR
		453	NR	NR	NR	453	2	NR
Wolchok, 2017 [14]	Melanoma	314	Not reached	NR	NR	313	2	NR
		316	37.6(29.1-not reached)	NR	NR	313	1	NR
		315	19.9(16.9–24.6)	NR	NR	311	1	NR
D’angelo, 2018 [15]	Sarcoma	43	10.7(5.5–15.4)	NR	NR	42	0	8
		42	14.3(9.6-not reached)	NR	NR	42	0	11
Long, 2018 [16]	Melanoma	35	NR	NR	NR	35	0	16
		25	NR	NR	NR	25	0	1
		16	NR	NR	NR	16	0	2
Motzer, 2015 [19]	Renal cancer	60	18.2	NR	NR	59	0	NR
		54	25.5	NR	NR	54	0	NR
		54	24.7	NR	NR	54	0	NR
Hamanishi, 2015 [18]	Ovarian cancer	20	20.0	NR	NR	20	0	5
Hida, 2017 [29]	Lung cancer	35	16.3(12.4–25.4)	NR	NR	35	0	3
Kudo, 2017 [30]	Esophageal cancer	65	10.8(7.4–13.3)	NR	NR	65	0	10
Maruyama, 2017 [31]	Hodgkin lymphoma	17	NR	NR	NR	17	0	6
Nishio, 2017 [32]	Lung cancer	76	17.1(13.3–23.0)	NR	NR	76	0	15
Overman, 2017 [33]	Colorectal cancer	74	NR	NR	NR	74	0	9
Rizvi, 2015 [34]	Lung cancer	117	8.2(6.1–10.9)	NR	NR	117	2	NR
Yamazaiki, 2017 [35]	Melanoma	24	NR	NR	NR	24	0	4
Younes, 2016 [36]	Hodgkin lymphoma	80	NR	NR	NR	80	0	5

*Abbreviation: G/GJC* gastric and gastro-esophageal junction cancer *FAE,* fatal adverse event *SAE* Serious adverse event*; OS* overall survival*; CI* confidence interval*; HR* hazard ratio*; NR* not reported

### Statistical analysis

To calculate the incidence, the number of patients receiving nivolumab and the number of SAEs/FAEs were extracted from eligible studies. For the OR calculations, patients treated with nivolumab were compared with those assigned to a chemotherapy/placebo arm in the same trial. Four studies [13–16] were not included in the OR analysis because ipilimumab was administered in the control arms. When the trials reported no SAE/FAE in one arm, a classic half-integer continuity correction was used for the calculation.

Statistical heterogeneity across the trials was evaluated by Cochran’s Q statistic. The *I*^*2*^ statistic was calculated to assess the extent of inconsistency attributable to the heterogeneity across different studies [17]. The assumption of homogeneity was considered invalid for *I*^*2*^ > 25% or *P* < 0.05. The pooled ORs and incidences were calculated using a fixed-effects model or a random-effects model, depending on the heterogeneity of the included trials. To check the impact of various clinicopathological variables on FAEs, we further conducted post hoc subgroup analysis based on the underlying malignancy and clinical phase.

All analyses were conducted using MedCalc 13.0 (MedCalc Software, Belgium) and Stata 12.0 (StataCorp, USA). Two-sided *P* < 0.05 were considered statistically significant.

## Results

### Search results and study characteristics

A total of 4175 potentially relevant articles were identified from the initial search, including 2140 studies from PubMed and 2035 trials from Embase. A total of 1982 articles were excluded because of duplications. After the titles and abstracts were screened, 2086 studies did not meet the inclusion criteria. Upon further review of the whole texts of the remaining 107 potentially eligible articles, 21 trials (36 arms) with 6173 patients were enrolled for the final analysis (Fig. 1).

Within these 21 eligible studies, 9 were phase 3 RCTs, 3 were phase 2 RCTs, and 9 were single-arm phase 2 trials. Of the 36 arms, 7 arms were with chemotherapy/placebo, 3 arms were with nivolumab + ipilimumab, and 2 arms were with ipilimumab (Table 1), leaving a total of 24 arms of patients to be evaluated for the incidence of SAEs/FAEs with nivolumab. The number of patients per nivolumab arm with associated safety data ranged from 16 to 453 (average of 156), with a total of 3732 patients with SAE/FAE information available (Table 2). The most common cancer types were lung cancer (6 studies, 1623 patients) and melanoma (5 studies, 2366 patients). The dosage of nivolumab was 3 mg/kg every 14 days in all but two studies. In one trial, the applied dosage was 1 or 3 mg/kg [18], and dosages of 0.3, 2 and 10 mg/kg were applied in another trial [19]. The treatment duration with nivolumab ranged from 1.5 months to 11.9 months (average of 5.3 months).

### SAEs

**Fig. 2:**
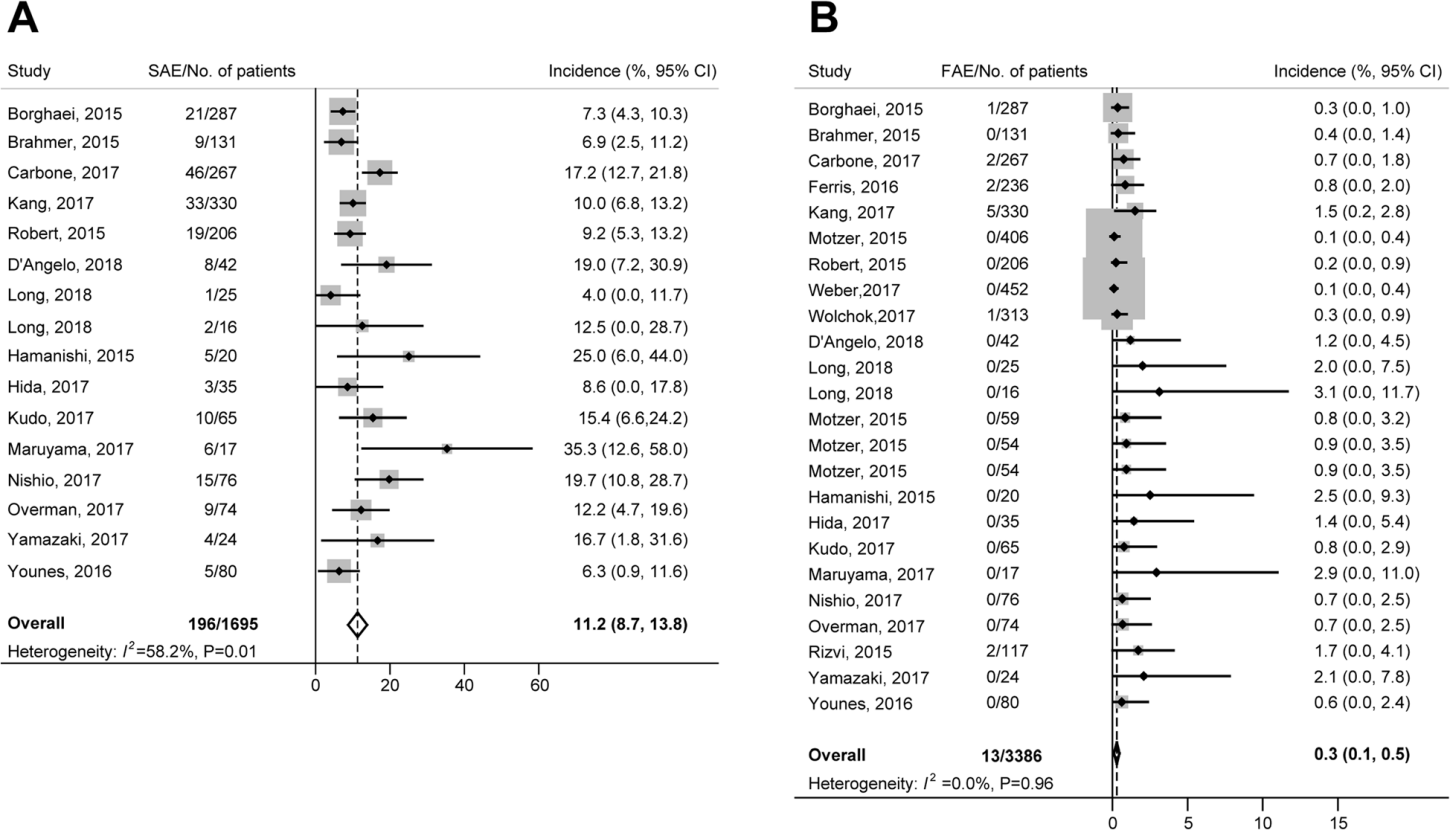
Overall incidence of SAEs (**a**) and FAEs (**b**) associated with nivolumab treatment FAE, fatal adverse event; SAE, serious adverse event

Of the 21 trials included in our study, SAE information was unavailable in 6 trials; therefore, 15 studies (16 arms) were eligible for the analysis of SAE incidence (Table 2). A total of 1695 subjects receiving nivolumab monotherapy were included, and 196 SAEs were reported. Using a random-effects model (significant heterogeneity, *I*^*2*^ = 58.2%; *P* = 0.01), the overall incidence of SAEs was 11.2% (95% CI, 8.7–13.8%; Fig. 2A). The incidence of SAEs varied significantly with cancer type (*P* < 0.001) and clinical phase (P < 0.001). The incidence rates for different cancer types were, in decreasing order, ovarian cancer (25.0%), sarcoma (19.1%), colorectal cancer (12.2%), lung cancer (11.8%), Hodgkin lymphoma (11.3%), gastric/gastro-esophageal cancer (10.9%) and melanoma (9.6%). The causes of nivolumab-related SAEs are summarized in Table 3. The most common SAEs occurred in the respiratory (*n* = 42, 21.4%), gastrointestinal (*n* = 15, 7.7%), and hepatic systems (*n* = 13, 6.6%). The most common SAEs were pneumonitis (*n* = 16, 8.2%), interstitial lung disease (*n* = 11, 5.6%) and colitis (*n* = 7, 3.6%). It was noted that pneumonitis occurred in lung cancer (*n* = 14) and gastric or gastro-esophageal junction cancer (*n* = 2); interstitial lung disease occurred in lung cancer (*n* = 6), gastric or gastro-esophageal junction cancer (*n* = 4) and Hodgkin lymphoma (n = 1); and colitis occurred in lung cancer (*n* = 3), gastric or gastro-esophageal junction cancer (*n* = 2), colorectal cancer (*n* = 1) and melanoma (*n* = 1).

**Table 3:** Specific causes for nivolumab-related SAE (serious adverse event) and FAE (fatal adverse event)

Nivolumab-related AE	SAE (*n* = 196)	Disease type (n)	FAE(*n* = 13)
Respiratory events	**42(21.4%)**	**LC (30), G/GJC (9), HL (1), M (1), S (1)**	**5(38.5%)**
Pneumonitis	16	LC (14), G/GJC (2)	LC (2), G/GJC (1), HNC (1)
Interstitial lung disease	11	LC (6), G/GJC (4), HL (1)	0
Pleural effusion	4	LC (2), M (1), S (1)	0
Dyspnea	2	LC (1), G/GJC (1)	G/GJC (1)
Lung disorder	2	LC (2)	0
Lung infection	2	G/GJC (2)	0
Bronchitis	1	LC (1)	0
Chronic obstructive pulmonary disease	1	LC (1)	0
Hypoxia	1	LC (1)	0
Respiratory tract infection	1	LC (1)	0
Pulmonary embolism	1	LC (1)	0
Gastrointestinal events	**15(7.7%)**	**LC (7), CC (3), G/GJC (2), S (2), M (1)**	**0(0.0%)**
Colitis	7	LC (3), G/GJC (2), CC (1), M (1)	0
Diarrhea	3	CC (1), LC (1), S (1)	0
Nausea	2	LC (2)	0
Decreased appetite	1	LC (1)	0
Anorexia	1	S (1)	0
Gastritis	1	CC (1)	0
Hepatic events	**13(6.6%)**	**LC (9), G/GJC (1), CC (1), HL (1), M (1)**	**1(7.7%)**
AST increased	6	LC (6)	0
Hepatotoxicity	5	LC (2), M (1), G/GJC (1), HL (1)	0
ALT increased	1	CC (1)	0
Transaminases increased	1	LC (1)	0
Hepatitis	0	NA	G/GJC (1)
Renal and urinary events	**9(4.6%)**	**LC (3), G/GJC (3), CC (1), HL (1), M (1)**	**0(0.0%)**
Blood creatinine increased	2	LC (2)	0
Hyponatremia	2	G/GJC (1), HL (1)	0
Acute kidney injury	1	CC (1)	0
Renal impairment	1	M (1)	0
Tubulointerstitial nephritis	1	LC (1)	0
Urinary tract infection	2	G/GJC (2)	0
Cardiovascular events	**6(3.1%)**	**LC (5), OC (1)**	**2(15.4%)**
Cerebrovascular accident	2	LC (2)	0
Atrial fibrillation	1	LC (1)	0
Cardiac tamponade	1	LC (1)	0
Deep vein thrombosis	1	OC (1)	0
Pericardial effusion	1	LC (1)	0
Cardiac arrest	0	NA	G/GJC (1)
Ischemic stroke	0	NA	LC (1)
Nervous system events	**6(3.1%)**	**M (2), LC (2), OC (2)**	**1(7.7%)**
Encephalitis	1	LC (1)	LC (1)
Headache	2	M (2)	0
Disorientation	1	OC (1)	0
Dizziness	1	LC (1)	0
Gait disorder	1	OC (1)	0
Endocrine events	**6(3.1%)**	**LC (2), G/GJC (2), CC (1), HL (1)**	**0(0.0%)**
Adrenal insufficiency	2	LC (1), CC (1)	0
Diabetic ketoacidosis	2	G/GJC (2)	0
Diabetes mellitus	1	HL (1)	0
Hypothyroidism	1	LC (1)	0
Musculoskeletal events	**5(2.6%)**	**LC (3), CC (2)**	**0(0.0%)**
Arthritis	1	CC (1)	0
Myasthenic syndrome	1	LC (1)	0
Osteonecrosis	1	LC (1)	0
Polymyalgia rheumatica	1	LC (1)	0
Pain	1	CC (1)	0
Blood events	**2(1.0%)**	**S (2)**	**1(7.7%)**
Anemia	1	S (1)	0
Decreased platelet count	1	S (1)	0
Neutropenia	0	NA	M (1)
Skin and subcutaneous tissue events	**1(0.5%)**	**HL (1)**	**0(0.0%)**
Rash	1	HL (1)	0
Other	**20(10.2%)**	**LC (6), G/GJC (5), HL (3), OC (2), S(2), M (1), CC (1)**	**3(23.1%)**
Pyrexia	5	LC (2), G/GJC (2), HL (1)	0
Infusion related reaction	4	LC (2), HL (2)	0
Fever	3	OC (2), S (1)	0
Dehydration	3	G/GJC (2), S (1)	0
Chills	1	LC (1)	0
Fatigue	1	G/GJC (1)	0
Radio-necrosis	1	M (1)	0
Stomatitis	1	CC (1)	0
Subdural hematoma	1	LC (1)	0
Multiorgan failure	0	NA	LC (1)
Hypercalcemia	0	NA	HNC (1)
Unknown reason	0	NA	G/GJC (1)

*Abbreviation: ALT* alanine aminotransferase*; AST* aspartate aminotransferase*; CC* colorectal cancer*; G/GJC* gastric or gastro-esophageal junction cancer*; HL,* Hodgkin lymphoma*; HNC* head and neck cancer*; LC* lung cancer*; M* melanoma*; OC* ovarian cancer*; S* sarcoma

**Fig. 3:**
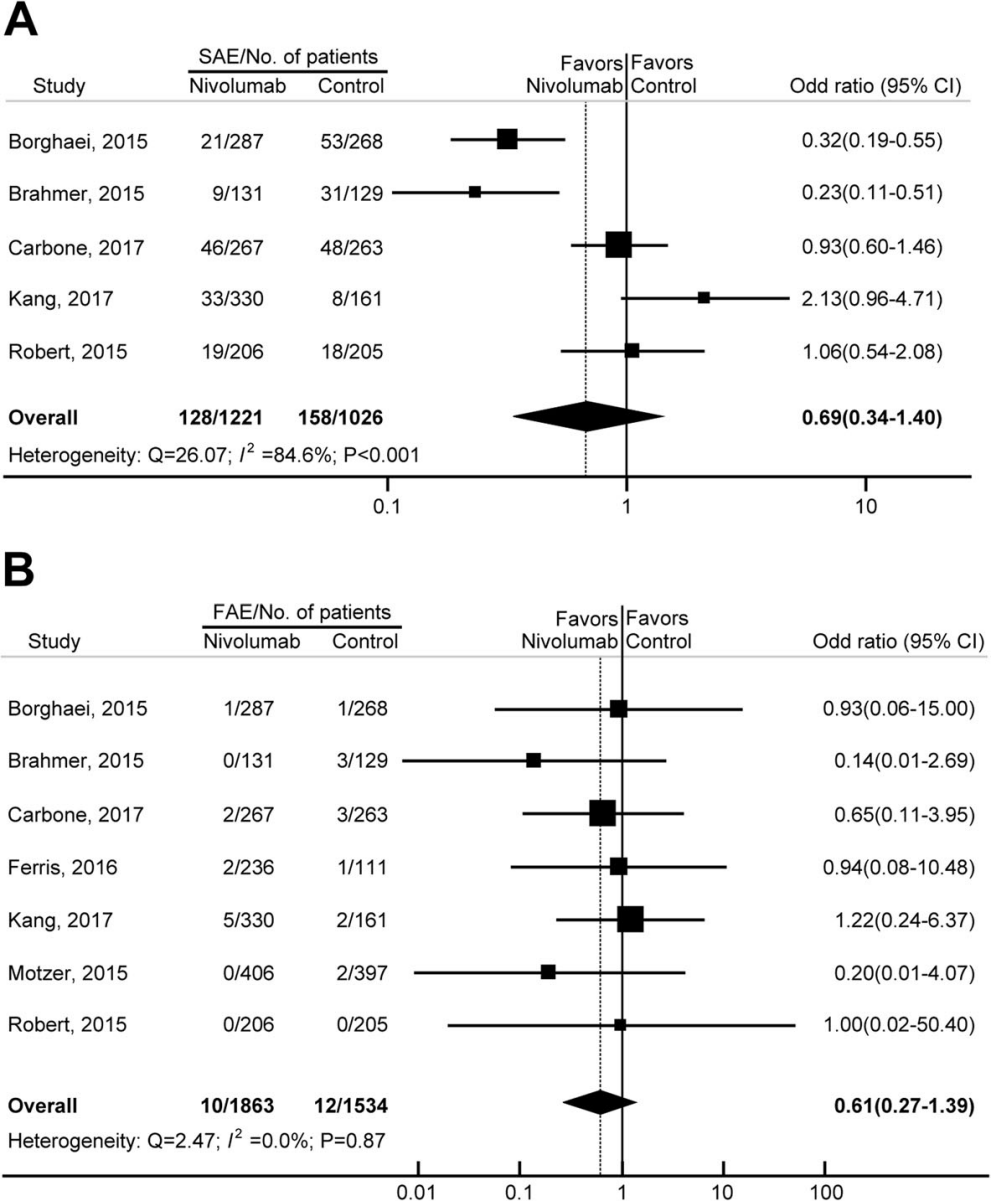
Odds ratios (ORs) of SAEs (**a**) and FAEs (**b**) associated with nivolumab versus the controls

Of the 15 eligible studies, 8 trials were single-arm phase 2 studies, 2 trials included other immunotherapy as controls, and the 5 remaining studies were eligible for OR analysis. Among the 2247 patients (nivolumab: 1221; control: 1026) in these five RCTs, the overall OR of SAEs induced by nivolumab was 0.69 (95% CI, 0.34–1.40, *P* = 0.29; incidence 10.5% versus 15.40%; Fig. 3A), indicating no significantly increased risk of SAEs associated with nivolumab compared with the controls. This estimate was obtained using a random-effects model because a significant heterogeneity in the increased risk of SAEs with nivolumab treatment was revealed (Q = 26.07, *I*^*2*^ = 84.6%, *P* < 0.001). The cause for this heterogeneity was explored, and the OR of SAEs with nivolumab differed significantly by cancer type (*P* < 0.01). The risk for SAEs for different tumor types were, in decreasing order, gastric/gastro-esophageal cancer (OR, 2.13; 95% CI, 0.96–4.71); melanoma (OR, 1.06; 95% CI, 0.54–2.08) and lung cancer (OR, 0.43; 95% CI, 0.18–1.02).

### FAEs

In this study, 3386 cancer patients receiving nivolumab from 21 trials (24 arms) were included in the analysis of the incidence of FAEs. A total of 13 FAEs were reported. Using a fixed-effects model, the overall incidence of FAEs was 0.3% (95% CI, 0.1–0.5%; Fig. 2B). No significant heterogeneity was observed (heterogeneity test, *I*^*2*^ = 0.0%; *P* = 0.96). The incidence rates for various tumor types were, in decreasing order, gastric/gastro-esophageal cancer (1.27%), head and neck cancer (0.85%), lung cancer (0.55%) and melanoma (0.09%). The causes of nivolumab-related FAEs were 4 cases of pneumonia and one case each of encephalitis, multiorgan failure, hypercalcemia, hepatitis, cardiac arrest, exertional dyspnea, ischemic stroke, neutropenia, and unknown reason (Table 3).

Of the 21 eligible studies, 9 trials were single-arm phase 2 studies, 5 trials included other immunotherapy as controls, and the remaining 7 studies were eligible for OR analysis. Among the 3397 patients (nivolumab: 1863; control: 1534) in the 7 eligible RCTs, the overall OR of FAEs induced by nivolumab was 0.61 (95% CI, 0.27–1.39, *P* = 0.24; incidence 0.5% versus 0.8%; Fig. 3B), indicating that the risk of nivolumab-related FAEs was not significantly different from those in the control arms. No significant heterogeneity was identified, despite clear disparities in cancer type, treatment duration and control type (Q = 2.47; *I*^*2*^ = 0.0%; *P* = 0.87). Because no significant heterogeneity was observed, subgroup analyses were not conducted for FAEs. To account for any potential clinical heterogeneity not detected by our statistical tests, we also pooled the data using a random-effects model, and the OR and 95% CI remained unchanged.

## Discussion

To our knowledge, this is the first study focused specifically on SAEs and FAEs among cancer patients treated with nivolumab. Based on 21 trials with 6173 patients, our results showed that the overall incidence of treatment-related SAEs and FAEs were 11.2% and 0.3%, respectively. SAEs/FAEs occurred in the major organ systems in a dispersed manner, with the most common toxicities appearing in the respiratory, gastrointestinal, and hepatic systems. Additionally, nivolumab administration did not increase the risk of SAEs/FAEs in adult patients with solid tumors. These findings demonstrated that nivolumab was a relatively safe antitumor agent and, therefore, should have clinical implications.

Immune checkpoint inhibitors (ICIs) have dramatically improved the outlook of cancer treatment. It is also well established that these agents are associated with immune-related AEs, which can be fatal in some cases. It was reported that ipilimumab could increase the risk of mortality by 130% in cancer patients, with an overall FAE incidence of 1.13% [5]. Interestingly, thus far, numerous patients have received nivolumab therapy, and immune-related toxicities similar to those associated with anti-CTLA-4 have been observed; however, in general, the frequency was generally lower with nivolumab. It is possible that because the PD-1 and PD-L1 checkpoint acts later in the T cell response, which results in a more restricted T cell reactivity toward tumor cells, nivolumab is well-tolerated in clinical practice [20]. In addition, it should be noted that most of the ipilimumab studies were performed relatively early, when the knowledge about immune-related AEs was lower and the management guidelines for such AEs were not established.

Previous research revealed that nivolumab treatment had a significantly lower risk for any grade of AE compared with chemotherapeutics, and the most common any-grade AEs were fatigue, rush, pruritus, diarrhea, nausea and asthenia [21]. Fatigue alone occurred in over 25% of cancer patients treated by nivolumab. In contrast, our analysis demonstrated that nivolumab treatment and conventional therapy had similar risks for severe toxicities in terms of SAEs and FAEs. Nivolumab-related SAEs occurred in 10% patients and were dispersed in the major organ systems. The incidence of serious fatigue was much lower in patients treated with nivolumab (*n* = 1, 0.06%) because of early detection and proper management.

Our study shows that the most common SAEs were pneumonitis, interstitial lung disease, and colitis. The higher risk of both all-grade and high-grade pneumonitis with ICIs has been reported by several studies [22–24]. Moreover, we also revealed that nivolumab-induced pneumonitis was the most common cause of FAEs. The pooled incidences of all-grade and high-grade pneumonitis in patients treated with PD-1/PD-L1 inhibitors were 3.2% and 1.1%, respectively [24], while our data revealed that the incidence of serious nivolumab-related pneumonitis was 0.9%, and the incidence of FAEs caused by pneumonitis was 0.2%. Pulmonary toxicity constitutes the predominant nivolumab-related cause of mortality. Clinicians should pay attention to any patient presenting with pulmonary symptoms, including hypoxia, dry cough, and shortness of breath. Pathologically, treatment-related immune pneumonitis was believed to be similar to interstitial pneumonitis associated with collagen vascular disease. Radiologically, evidence of interstitial pneumonitis may be presented using high-resolution computed tomography [23].

Immune-related colitis is also an increasingly encountered SAE requiring appropriate management. However, a recent meta-analysis revealed that the incidence of colitis induced by immune checkpoint inhibitors was higher in ipilimumab-containing regimens compared with PD-1/PD-L1 inhibitors [25]. The incidences of all-grade and high-grade colitis in patients treated with PD-1/PD-L1 were 1.3% and 0.9%, respectively [25], while our data demonstrated that the incidence of serious colitis was 0.4%.

The prevention of nivolumab-related SAEs/FAEs consists of early detection and aggressive treatment of potentially dangerous AEs, such as pneumonitis, colitis and hepatotoxicity. General management principles include temporary or permanent application of nivolumab. Moreover, the administration of immune modulatory drugs, like infliximab, glucocorticoids and azathioprine, has proved helpful in many studies [26, 27].

Our study has important clinical implications. It is reported that mortalities associated with adverse drug reactions account for approximately 5% of all hospital fatalities [28]. Accordingly, benefit/risk evaluation should play an essential role in the decision-making process during the selection of cancer treatments. Patients should recognize the increased risk of treatment-related mortality before consenting to any therapy. Our study could be important in considering the benefit/risk trade-off by providing the overall incidences and relative risks for SAEs/FAEs in patients treated with nivolumab.

Our study has several strengths. We performed a comprehensive review, utilizing the most up-to-date published data. All of the included original studies are phase 2 or phase 3 RCTs, which minimized selection bias. Moreover, with the accumulating evidence and enlarged sample sizes, this study had enhanced statistical power, leading to more reliable and precise clinical outcome estimates.

This study also has some limitations. First, our study is based on data from clinical trials rather than individual patients. This may include some confounding factors, such as previous therapies received, patients’ comorbidities, and concomitant medications. Second, clinical trials are usually not designed specifically to address toxic events; thus, asymptomatic adverse events may be ignored in the prospective assessment. Third, the incidence of SAEs among the eligible trials had significant heterogeneity. In this study, we adjusted this heterogeneity by applying a random-effects model to calculate the overall incidence.

## Conclusions

In summary, nivolumab treatment did not increase the risk of serious and fatal drug-related adverse events. Considering that immune checkpoint inhibitors have shown important clinical benefit in numerous cancers, nononcologists should be advised of their potential adverse events. Additionally, future studies are needed to identify patients at high risk for SAEs/FAEs to aid in the development of optimal monitoring strategies and the exploration of treatments to decrease risks.

## Abbreviations

ALT: alanine aminotransferase
AST: aspartate aminotransferase
CC: colorectal cancer
CI: confidence interval
CTLA-4: cytotoxic T-lymphocyte-associated protein 4
FAE: fatal adverse event
G/GJC: gastric or gastro-esophageal junction cancer
HL: Hodgkin lymphoma
HNC: head and neck cancer
HR: hazard ratio
ICI: immune checkpoint inhibitor
LC: lung cancer
M: melanoma
NR: not reported
OC: ovarian cancer
OS: overall survival
PD-1: programmed cell death protein 1
PD-L1: programmed death-ligand 1
S: sarcoma
SAE: Serious adverse event
