# Correction to: Serious adverse events and fatal adverse events associated with nivolumab treatment in cancer patients

**DOI:** 10.1186/s40425-018-0487-7

**Published:** 2019-01-11

**Authors:** Bin Zhao, Hong Zhao, Jiaxin Zhao

**Affiliations:** 1 The Second Affiliated Hospital & Yuying Children’s Hospital, Wenzhou Medical University, Wenzhou, China; 2 The Third Affiliated Hospital of Harbin Medical University, Harbin, China; 3 The Fourth Affiliated Hospital of Harbin Medical University, Harbin, China

**Correction to: Journal for ImmunoTherapy of Cancer (2018) 6:101**

**Doi 10.1186/s40425-018-0421-z**

Following publication of the original article [1], the authors reported an error in their listed affiliations.

In this Correction Dr. Jiaxin Zhao is related to two affiliations:

1. The Second Affiliated Hospital & Yuying Children’s Hospital, Wenzhou Medical University, Wenzhou, China.

3. The Fourth Affiliated Hospital of Harbin Medical University, Harbin, China.

The published apologizes for any inconvenience caused by this error.
